# Some Observations on the Use and Abuse of Opium

**Published:** 1915-09

**Authors:** M. T. Davis

**Affiliations:** Atlanta, Ga.


### SOME OBSERVATIONS ON THE USE AND ABUSE OF OPIUM.

M. T. Davis, M. D., Atlanta, Ga.

Inebriety is often spoken of as a disease, this being the general term, which may be specifically modified to suit the special narcotic for which the subject evinces an abnormal appetite, as for instance, Alcoholo-mania, Morphomania, and so on.

I think, however, that it is more appropriate to say that inebriety is that morbid condition of the nervous system which craves the intoxicating effects of narcotics. It is a defect in the nervous organization of the individual, which makes his existence under the influence of some narcotic intoxication more pleasurable, or less painful than during abstinence.

This craving may arise from the action of the narcotic upon the subject himself; it may come to him as a congenital fault, or it may be the outgrowth of nervous disturbances coming on later in life, coupled with certain influences of environment.

If we accept the foregoing as true, then there are many inebriates who have never tasted drink, nor used a grain of any drug. They are born inebriates, nature having endowed them with the inebriate temperament, and the only reason they have never become victims of drugs or drink is that they have lacked opportunity—their environment has protected them. Had they been thrown with bibulous associates or been the victims of a painful malady they would have yielded to the subtle, seductive influence of artificial stimulants.

Probably no man has ever deliberately, intentionally become addicted to the use of opium, nor even thought that HE could be so weak as to become thus enslaved. Yet, once in the grip of this monster, few, if any, have ever given it up alone and unaided, because of its destructive action upon the will.

The advent of the Hypo-Syringe has largely increased the use of opium in the form of morphine. The physician is, alas, too often culpable when through ignorance or laziness he is induced to administer morphine day after day for several weeks or months, often telling the patient what he is getting, eventuating in the NECESSITY for the continued daily use of the drug—the so-called morphine habit. This necessity is brought about by the cumulative action of opium, the drug being stored up in the nerve cells and tissues of the body, more rapidly than it is eliminated.

Opium, though classed as a narcotic sedative, is primarily a stimulant of the highest order. There comes a time, therefore, when the nerves begin to cry out for their accustomed pabulum to crave, and to demand the stimulant upon which they have by this time been taught to depend. This craving for opium is manifested in the suffering of the morphine addict, upon his attempting to do without, or even to materially lessen his accustomed dose. It begins as soon as the last dose has ceased to stimulate. This suffering is indescribable, but it is the very refinement of agony, causing great beads of sweat to stand out upon the brow of the patient really in need of morphine.

After a variable time, depending mainly upon the temperament of the individual, opium destroys that very self-control necessary in order to enable one, alone and unaided, to discontinue its use. So that while, when first begun, it may be spoken of as a habit, after it has been indulged in for any considerable length of time, and has begun to be stored away in the tissues, it brings about such pathological changes in the bodily structure, especially in the nervous system, as to constitute actual diseased conditions, one of the many symptoms of which is loss of self-control or will-power—so that the man is no longer master of his own actions—they are completely dominated by opium. The will is present, but to perform, he is powerless. This is at first true as regards opium only, but soon his whole life is tainted by the influence of the drug, and procrastination marks his every undertaking, putting off from day to day the most vitally important as well as the most trivial things.

Strange irony of fate it is that at about the same time he begins to realize that he cannot do without morphine, that drug ceases to produce any pleasureable sensations, or even to relieve pain except when used in largely increased quantities. So that contrary, to the general belief, the unfortunate victim continues its use, not because of any pleasure derived therefrom, but solely in order to prevent the suffering that would ensue did he not take it.

Individuals of the inebriate class frequently show signs early in life which are characteristic of this type. They form certain senseless silly habits, such as biting their nails, stammering, making facial contortions or grimaces. Often they suffer from convulsions in infancy, and from bed-wetting until the approach of puberty. Many are found to have adherent or redundant foreskins, and these nearly always masturbate for a period longer than does the average normal boy.

The day is near at hand when a child of this type will be recognized as a neurotic, and of the inebriate temperament, and one who will be more prone to yield to evil influences than his more phlegmatic brother: hence, he will have his weak points explained to him, and will be taught to guard and to protect them from assaults from without. Moreover, he will be trained from his infancy in lessons of self-control and self-denial. He will be taught that his weaknessses constitute no valid excuse for pampering himself or being pampered, since, if he will, he may by fighting, overcome his handicap and grow, perhaps slowly, but surely, into a strong manly man.

To take a patient off of morphine is but the beginning of a cure, and to turn him loose to assume the burdens and responsibilities of life before nature has had time to right the wrong done her, is but to invite an inevitable relapse. There's no fighting like it. It means watching every hour, every minute. Some days it is easy, some days it is hard, but it's never so easy that he can say there's no need to watch. In sleep it whispers and wakes him, in the morning it casts a shadow on his path—and one day when he thinks it has been conquered, and that he is master, up it springs with fury and the fight begins again. But he becomes stronger, and after many battles, when he has learned never to be off his guard, and to know by instinct where every ambush is, then at last the victory is his. But it is hard, it is bitter, and sometimes it seems hardly worth the struggle. But it is—it IS worth the struggle.

Fortunately we now have in the Hydrobromide of Hyoscine, a drug which immediately allays the craving for opium. Its antidotal effect upon the subjective sensations of one in need of opium is marvelous, and it has robbed the withdrawal period of the terrors it once held for the victim.

Given a patient using morphine, I administer mercurial purgative at bed time, and his customary dose of opiate. The next day I give him just half the quantity of drug he has been accustomed to taking, and in four doses, at 6, 10, 2 and 6, and each succeeding day reduce the previous day's dose by one-fourth, until he is taking but a grain in 24 hours. At this time he begins to feel the craving for his drug, which is at once allayed by combining with each dose of morphine 1-600 grain of Hyoscine. In this way we may continue to reduce each day's quantity by one-fourth, until, within ten days from the beginning we have withdrawn the drug entirely without any shock, suffering or other unfavorable results.

This is but the first step in effecting a cure, and the patient must be safeguarded by every means possible until his physical stamina and his morale as well, have been regained.

Few cigarette smokers are ever permanently cured unless they abandon that habit also; since they seem to cause just enough stimulation to act as an irritant to the hypersthetic nerves, inducing a craving for a stronger stimulant.

I have omitted the sypmtomalology of the opium habit, since it is more or less familiar to us all, and the pathology for the reason that we know so little about it. After being restored to physical health, the patient must be treated morally by appealing to the best that is in him and making him feel that he still has our confidence. In this way whatever of manhood he has left will reassert itself and he will thus have his own opportunity for restoration to a life of health, happiness and usefulness.

